# Low-dose exposure to bisphenol A in combination with fructose increases expression of genes regulating angiogenesis and vascular tone in juvenile Fischer 344 rat cardiac tissue

**DOI:** 10.1080/03009734.2016.1225870

**Published:** 2016-09-13

**Authors:** Helén Klint, Margareta H. Lejonklou, Elina Karimullina, Monika Rönn, Lars Lind, P. Monica Lind, Eva Brittebo

**Affiliations:** a Uppsala University, Department of Pharmaceutical Biosciences, SE-75124 Uppsala, Sweden;; b Uppsala University, Department of Medical Sciences, SE-75185 Uppsala, Sweden;; c University of California, Irvine, Department of Developmental and Cell Biology, Irvine, CA 92697, USA

**Keywords:** Angiogenesis, bisphenol A, cardiomyocytes, cardiovascular disease, endocrine disruption, fructose, heart, vascular tone

## Abstract

**Objectives:**

Epidemiological studies report associations between exposure to the high-volume chemical and endocrine disruptor bisphenol A (BPA) and cardiovascular disorders, but there is a lack of experimental studies addressing the mechanisms of action of BPA on the cardiovascular system. In the present study, effects on markers for cardiovascular function of exposure to BPA and fructose *in vivo* in rat cardiac tissues, and of BPA exposure in human cardiomyocytes *in vitro*, were investigated.

**Materials:**

Juvenile female Fischer 344 rats were exposed to 5, 50, and 500 μg BPA/kg bodyweight/day in their drinking water from 5 to 15 weeks of age, in combination with 5% fructose. Further, cultured human cardiomyocytes were exposed to 10 nM BPA to 1 × 10^4^ nM BPA for six hours. Expression of markers for cardiovascular function and BPA target receptors was investigated using qRT-PCR.

**Results:**

Exposure to 5 μg BPA/kg bodyweight/day plus fructose increased mRNA expression of *Vegf*, *Vegfr2*, *eNos*, and *Ace1* in rat heart. Exposure of human cardiomyocytes to 1 × 10^4^ nM BPA increased mRNA expression of *eNOS* and *ACE1*, as well as *IL-8* and *NFκβ* known to regulate inflammatory response.

**Conclusions:**. Low-dose exposure of juvenile rats to BPA and fructose induced up-regulation of expression of genes controlling angiogenesis and vascular tone in cardiac tissues. The observed effects of BPA in rat heart were in line with our present and previous studies of BPA in human endothelial cells and cardiomyocytes. These findings may aid in understanding the mechanisms of the association between BPA exposure and cardiovascular disorders reported in epidemiological studies.

## Introduction

A growing bulk of scientific evidence has raised concerns about the health effects of human exposure to the endocrine disruptor bisphenol A (BPA). BPA is a key component in polycarbonate plastics and epoxy resins, which are widely used in the manufacturing of consumer products (1). Oral exposure through consumption of plastic-packaged and canned foods and beverages has been identified as the main route of exposure, but other possible exposure sources, such as dermal exposure from thermal papers, have also been recognized (2).

According to a systematic review of studies on BPA, human exposure to this hormone-disrupting contaminant is low but widespread (3). Orally ingested BPA is rapidly eliminated via glucuronidation in the liver. However, absorption from the oral cavity leads to much higher internal exposure of BPA than that absorbed from the gastrointestinal tract (4). A review of more than 80 biomonitoring studies report that unconjugated and conjugated BPA is routinely detected in human biological samples, indicating that the general population is internally exposed to the unconjugated form of BPA (5).

The reported serum concentrations of unconjugated BPA were previously believed to be below the levels that can activate the classical estrogen receptors, estrogen receptor α (ERα) and estrogen receptor β (ERβ), in experimental model systems, and that it thus would be unlikely that environmental exposure to BPA would cause adverse effects in humans and animals by a direct effect of unconjugated BPA on ERα and ERβ (6). However, recent studies show that the mechanisms by which BPA causes biological effects are complex and involve both genomic and non-genomic pathways elicited by a variety of intracellular receptors (7). The nuclear estrogen-related receptor γ (ERRγ) and the membrane-bound estrogen receptor G-protein-coupled receptor 30 (GPR30) are, aside from ERα and ERβ, the receptors that mainly have been associated with the effects of BPA. However, other receptors such as the thyroid receptor and the aryl hydrocarbon receptor control the expression of a number of genes relevant in immune cell subsets, and are thus potentially also important for cardiovascular function, and may also be involved in BPA’s mechanism of action (8–10). Further, BPA has been described to be a selective estrogen receptor modulator (SERM), which implies that the modes of action can vary depending on age, sex, as well as across different tissue and cell types (11). Thus, environmental exposure levels of BPA may have direct or indirect effects on the cardiovascular system, by yet unknown mechanisms, and expression of various BPA target receptors in cardiovascular tissues may be a determinant factor for the effects of BPA in the cardiovascular system (12). Recent experimental findings indicate that the lifelong exposure to BPA impacts cardiac structure and function in mice (13). Eight weeks of BPA exposure causes myocardial injury in hyperlipidemic rabbits (14), and chronic exposure to BPA results in cardiac remodeling, atherosclerosis, and altered blood pressure in rodents (15).

A few large epidemiological studies suggest that high levels of exposure to BPA are associated with an increased risk for cardiovascular disease. Two cross-sectional studies of the National Health and Nutrition Surveys (NHANES) 2003/06 cohort and one prospective study of the European Prospective Investigation of Cancer–Norfolk UK cohort report associations between elevated urinary BPA and an increased incidence of cardiovascular disease (16–18). Furthermore, endothelial dysfunction has been described as an adverse effect of BPA in children (19).

We have previously reported that 1 nM–1 μM BPA increased the mRNA expression of factors that regulate angiogenesis and vascular tone: endothelial nitric oxide synthase (*eNOS/NOS3*), vascular endothelial growth factor A (*VEGF*), VEGF receptor 2 (*VEGFR2*), and angiotensin I converting enzyme (*ACE1*) in primary human endothelial cells *in vitro* (20). VEGF and ACE1 are known as estrogen-responsive genes in cardiovascular cells and tissues (21,22). VEGF exerts its influence by targeting the VEGFR2 in endothelial cells. This triggers a signaling cascade including eNOS that ultimately leads to increased production of nitric oxide, stimulating angiogenesis, increased vascular permeability, and inflammation in the vasculature (23). In adults, low levels of VEGF are expressed in all vascularized tissues to maintain vascular homeostasis (24). Increased VEGF signaling and angiogenesis are involved in early plaque formation (25), and VEGF-driven angiogenesis is increasingly being recognized as important in the progression and rupture of atherosclerotic plaques (26). ACE1 is responsible for the formation of angiotensin II, which causes vasoconstriction leading to increased blood pressure and is strongly implicated in the development of cardiovascular disease (27,28).

The perturbations preceding BPA-induced vascular effects remain to be elucidated in order to get a better understanding of the BPA-induced pathology. In a recent report on rat cardiac tissue from the same study as the present study, it was demonstrated that the heart proteome was altered following BPA and fructose exposure (29). Fructose was added to mimic a modern dietary pattern associated with the development of cardiovascular disease (30).

The primary objective of this study was to investigate if long-term exposure to low doses of BPA and fructose could also change biomarkers for cardiovascular function, inflammation, and BPA target receptors in rat cardiac tissue. Furthermore, the effects of BPA on markers for cardiovascular disease were examined in human cardiomyocytes.

## Materials and methods

### Chemicals

BPA: (CH_3_)_2_C(C_6_H_4_OH)_2_, CAS nr. 80-05-7, ≥99% purity, and fructose: C_6_H_12_O_6_, CAS nr. 57-48-7, ≥99% purity, were purchased from Sigma-Aldrich (St. Louis, MO, USA).

### Maintenance and exposure of animals

The rat hearts examined in the present study originate from a study on effects of BPA and fructose on obesity, infiltration of liver fat, and levels of circulating APO A-1 (31). In this study 60 female Fischer rats (F344) were purchased from Charles River International (Salzfeld, Germany) at 3 weeks of age and housed at an Uppsala University animal facility. Rats were maintained on standard pellet RM1 diet from NOVA-SCB (Sollentuna, Sweden) and water *ad libitum* in a temperature-controlled and humidity-controlled environment with a 12-hour light/dark cycle. To minimize background BPA exposure animals were housed in non-polycarbonate cages (Eurostandard IV), three animals per cage, and glass water bottles were used. Animals were acclimatized for 2 weeks prior BPA exposure. To mimic the route of human exposure, rats were exposed to BPA via their drinking water in combination with a modest dose of fructose (5%). The rats were randomly assigned to five groups; water control, fructose control, low-dose (5 μg/kg bodyweight/day) BPA, medium-high-dose (50 μg/kg bodyweight/day) BPA, and high-dose (500 μg/kg bodyweight/day) BPA; the latter three groups in combination with fructose (Table 1).

**Table 1.** Exposure of rats to BPA from 5 to 15 weeks of age.

	Exposure indrinking water		
Group	μg/mL BPA	% fructose	Mean BPA exposure(μg/kg bodyweight/day)
Water control	vehicle^a^	0	0
Fructose control	vehicle	5	0
Low-dose BPA	0.025	5	5.0
Medium-high-dose BPA	0.25	5	53.5
High-dose BPA	2.5	5	476

^a^1% ethanol in the drinking water was used as vehicle control.

Rats were exposed to BPA in their drinking water from 5 to 15 weeks of age as described in Table 1. BPA was dissolved in ethanol, and the stock solutions were then diluted 1:100 in their drinking water to reach the final exposure concentrations; 1% ethanol in their drinking water was used as a vehicle for the water and fructose controls. Food and water consumption and individual bodyweight was monitored weekly. There were no differences in food or water consumption between fructose controls and low-dose BPA, medium-high-dose BPA, or high-dose BPA. Weight gain during the study was similar in all treatment groups. After 10 weeks of exposure the rats were sacrificed by exsanguinations from the abdominal aorta under anesthesia with 90 mg/kg Ketalar (Pfizer, New York, NY, USA) and 10 mg/kg Rompun (Bayer, Leverkusen, Germany). Four rat heart samples/treatment group were randomly selected, dissected and stored in RNAlater (Qiagen, Hilden, Germany) at –20 °C for qRT-PCR analysis.

All animal experiments were approved by the Ethical Committee on Animal Experiments in Uppsala, approval number C329/09 and C29/11, following guidelines laid down by the European Union Legislation (Convention ETS123 and Directive 2010/63/EU).

### Cell culture and treatment of primary human cardiomyocytes

Fetal human cardiomyocytes (HCMs) were purchased from Science Cell (Carlsbad, CA, USA) and cultured according to the manufacturer’s instructions. HCMs were isolated, purified, characterized, and cryopreserved by the manufacturer and stored in liquid nitrogen until cell culture experiments. Briefly, HCMs were seeded in cardiomyocyte medium with 5% fetal bovine serum and 5.5 mM glucose on poly-L-lysine coated 6-well cell culture plates at a density of 5,000 cells/cm^2^. The cells were maintained at 37 °C in 5% CO_2_/95% air for 4 days before treatment with BPA; the cell culture media were changed after 2 days.

BPA was dissolved in dimethyl sulfoxide (DMSO), and the stock solutions were diluted 1:1,000 in cell culture medium directly before experiments. Experiments were performed on HCMs in the first passage when the cells had reached approximately 90% confluence. The cell culture medium was changed to an exposure medium containing 10 nM BPA, 1 × 10^3^ nM BPA, 1 × 10^4^ nM BPA, or vehicle (0.1% DMSO) in triplicate cell cultures per treatment and incubated for 6 hours. After 6 hours of incubation, the exposure medium was removed and the cells were lysed by the addition of 350 μL of RLT lysis buffer (Qiagen, Hilden, Germany). The cell lysates were stored at –20 °C.

### RNA purification of rat cardiac tissues and human cardiomyocytes

Cardiac biopsies were transferred from RNAlater to Eppendorf tubes in a liquid nitrogen bath and ground, using a chilled pestle. Complete homogenization of the tissue biopsies was accomplished by the addition of 350 μL lysis buffer (as described above) and sonication in an UCD-300 bioruptor (Diagenode, Denville, NJ, USA), 20 cycles 30 s on/off, on ice. RNA purification from cardiac tissue slices was performed using the RNeasy Mini Kit for fibrous tissue with the addition of DNase treatment for elimination of possible DNA contamination (Qiagen, Hilden, Germany). RNA purification from HCM lysates was performed using the RNeasy Mini Kit with the additional steps of shredder columns for complete homogenization and DNase treatment for elimination of possible DNA contamination (Qiagen).

### Quantitative RT-PCR

RNA integrity was analyzed with the Experion RNA StdSens Analysis Kit (Bio-Rad Laboratories, Hercules, CA, USA). RNA samples from four animals from each treatment group with an RNA integrity value >7 were used for qRT-PCR analysis. RNA concentration was determined using a Nanodrop ND-1000 (Bio-Rad Laboratories); cDNA synthesis from 1 μg of total RNA and qRT-PCR analysis was performed as previously described using TaqMan reverse transcription reagents (Applied Biosystems, Foster City, CA, USA) and IQ SYBR Green Supermix (Bio-Rad Laboratories) (32). Primer sequences are listed in Table 2. *Tbp*, *Gapdh*, and *Arbp* were employed as endogenous reference genes for analysis of mRNA expression in rat cardiac tissues. *ACTB*, *RPL13A*, and *TUBB* were employed as endogenous reference genes for analysis of mRNA expression in HCMs. The mean normalized expression (MNE) of the target genes in each sample was calculated based on the ratio between the target gene and the geometric mean of the reference genes (33,34). Threshold and cycle threshold values were automatically calculated for each sample by iCycler IQ software. The efficiency of the PCR reactions was determined using the publicly available software LinRegPCR as proposed by Ramakers and co-workers (35).

**Table 2.** The qRT-PCR primer sequences of genes included in the study.

Accession no.	Gene symbol	Forward primer sequence	Reverse primer sequence
NM_031836.2	*Vegfa*^a^	AAAAACGAAAGCGCAAGAAA	TTTCTCCGCTCTGAACAAGG
NM_001110333.1			
NM_001110334.1			
NM_001110335.1			
NM_001110336.1			
NM_013062.1	*Vegfr2*	GGAGATTGAAAGAAGGAACGAG	TGGTACATTTCTGGGGTGGT
NM_021838	*Nos3*	TGACCCTCACCGATACAACA	CGGGTGTCTAGATCCATGC
NM_012544.1	*Ace*	GGTCTGAGTACATCAACCTGGA	GTCTGTGCCCACATGTTCC
NM_134432	*Agt*	CACCTACGTTCACTTCCAAGG	AGAACTCATGGAGCCCAGTC
NM_012580	*Hmox*	GTCAAGCACAGGGTGACAGA	CTGCAGCTCCTCAAACAGC
NM_199267.2	*NFκβ*	AAGCCAGCACCCCAGCCCTA	GGATGGTGCCAGGGCCAAGG
NM_017232	*Ptgs2*	CTACACCAGGGCCCTTCC	TCCAGAACTTCTTTTGAATCAGG
NM_012589	*Il-6*	CCCTTCAGGAACAGCTATGAA	ACAACATCAGTCCCAAGAAGG
NM_012689.1	*Erα*	CGGAGGAGCCTAGCCAGAGCC	GAAGCCCTCTGCTTCCGGGGG
NM_012754.1	*Erβ*	GCATCCCTAGGCACCCAGGTC	AGCCGGACTCCCAGCCACTG
NM_133573	*Gpr30*	GCCATGGCTGCAACTACTCCAG	GAGGGCCAGAGGGGTGCTGT
NM_203336	*Errγ*	TCCCAGCAGCCTTTTGAACCCG	ATGACGGGGGAGTCTGGTGCT
NM_022402	*Arbp*	GGGCAATCCCTGACG	AGCTGCACATCGCTC
NM_002046	*Gapdh*	GGGCTCTCTGCTCCT	TCAGGTGAGCCCCAG
NM_001004198	*Tbp*	CCCACAGGGTGCCAT	ACGCAGTTGTTCGTG
NM_003376	*VEFGA*	TTAAACGAACGTACTTGCAGATG	GAGAGATCTGGTTCCCGAAA
NM_002253	*VEGFR2*	GAACATTTGGGAAATCTCTTGC	CGGAAGAACAATGTAGTCTTTGC
NM_000603	*NOS3*	TCTTCCTGGACATCACCTCC	CTTCCACTCCTCGTAGCGTC
NM_000789.3	*ACE*	GCCCGGCAACTTTTCTGCTGAC	AGCATGGCGTAGCTTCGCGA
NM_001122742.1	*ERα*	TTACTGACCAACCTGGCAGA	ATCATGGAGGGTCAAATCCA
NM_001437.2	*ERβ*	CGTCAGGCATGCGAGTAA	AAGCACGTGGGCATTCAG
NM_001505.2	*GPR30*	ACTGTGAAATCCGCAACCAT	GAGCTGCTCACTCTCTGGGTA
NM_001438.3	*ERRγ*	CACTGTCGCAGTTTGAAA GG	TGTGGTGGTTGACGCTGT
NM_012423	*RPL13A*	CTGGACCGTCTCAAGGTGTT	GCCCCAGATAGGCAAACTT
NM_178014	*TUBB*	TTAACCATGAGGGAA	CTGATCACCTCCCAG
NM_001101.3	*ACTB*	GAAAATCTGGCACCACACCT	TAGCACAGCCTGGATAGCAA

### Statistical analysis

Significant differences between the treatment groups (normal distribution) were determined using a generalized linear model. If the *P* value from the overall test of association was <.05, an LSD *post hoc* test was used for between-subjects analysis. The statistical analyses were performed using IBM SPSS^®^ software.

## Results

### Long-term BPA exposure increased the mRNA expression of Vegf, Vegfr2, Ace1, and eNos in rat cardiac tissue

**Figure 1.**
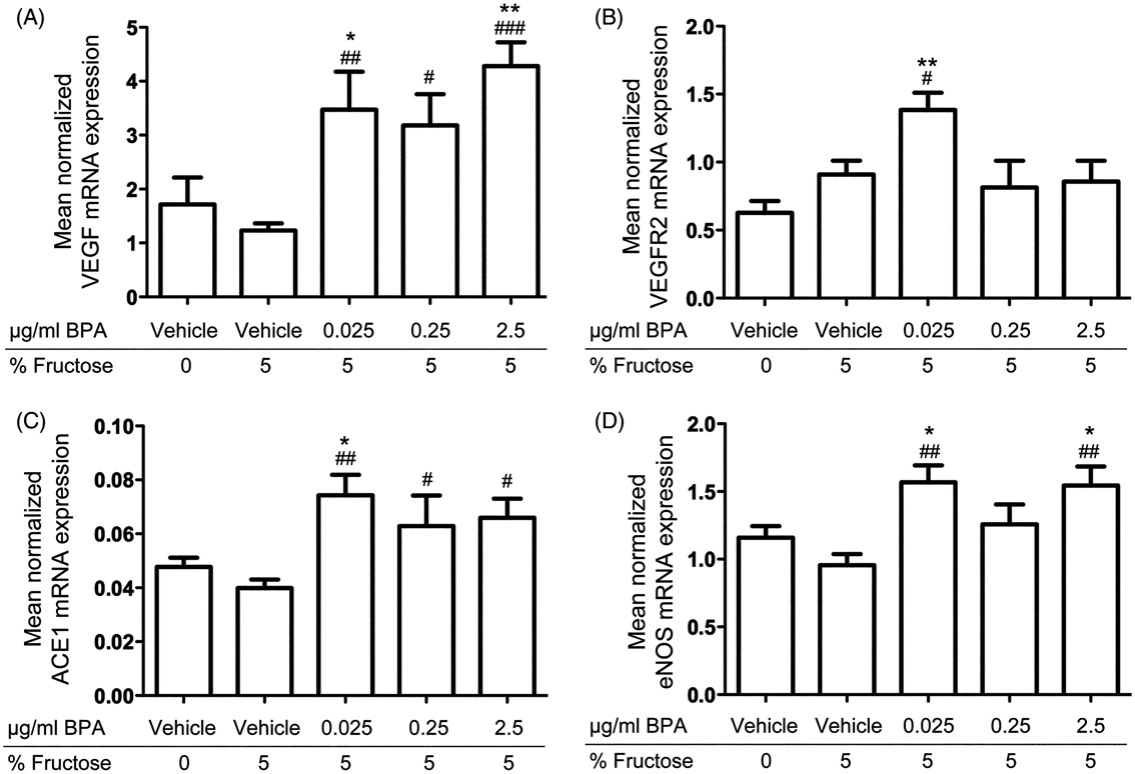
*In vivo* effects of long-term BPA exposure on *VEGF*, *ACE*, *eNOS*, and *VEGFR2* mRNA expressions in rat cardiac tissue. A: Rats exposed to 0.025 μg/mL, 0.25 μg/mL, and 2.5 μg/mL BPA showed increased mRNA expression of *VEGF* compared to fructose controls and water controls. B: *VEGFR2* mRNA expression was increased in rats exposed to 0.025 μg/mL BPA compared to fructose controls and water controls. C: *ACE1* mRNA expression was increased in rats exposed to 0.025 μg/mL, 0.25 μg/mL, and 2.5 μg/mL compared to fructose controls and in rats exposed to 0.025 μg/mL BPA compared to water controls. D: Rats exposed to 0.025 μg/mL and 2.5 μg/mL BPA showed increased *eNOS* mRNA expression compared to fructose controls and water controls. Each bar represents mean normalized mRNA expression ± SEM of four animals, each analyzed in three replicates. #*P* < .05, ##*P* < .01, ###*P* < .001, compared to fructose controls. *P < .05, **P < .01, ***P < .001, compared to water controls.

The qRT-PCR analysis revealed increased mRNA expression of *Vegf*, *Vegfr2*, *Ace1*, and *eNos* in cardiac tissue of rats exposed to BPA in their drinking water from preadolescence to adulthood compared to controls (Figure 1). The results showed increased *Vegf* mRNA expression in rats exposed to 5 μg BPA/kg bodyweight/day (*P* < .01), 50 μg BPA/kg bodyweight/day (*P* < .05), and 500 (*P <* .001) μg BPA/kg bodyweight/day compared to fructose controls; also, rats exposed to 5 μg BPA/kg bodyweight/day (*P <* .05) and 500 (*P <* .01) μg BPA/kg bodyweight/day showed increased *Vegf* mRNA expression compared to water controls (Figure 1(A)). The *Vegfr2* mRNA expression was increased in rats exposed to 5 μg BPA/kg bodyweight/day compared to fructose (*P <* .05) and vehicle controls (*P <* .01) (Figure 1(B)). Rats exposed to 5 μg BPA/kg bodyweight/day (*P <* .01), 50 μg BPA/kg bodyweight/day (*P <* .05), and 500 (*P <* .05) μg BPA/kg bodyweight/day showed increased *Ace1* mRNA expression compared to fructose controls, and rats exposed to 5 μg BPA/kg bodyweight/day also showed increased *Ace1* mRNA expression compared to water controls (*P <* .05) (Figure 1(C)). Rats exposed to 5 μg BPA/kg bodyweight/day and 500 μg BPA/kg bodyweight/day showed increased *eNos* mRNA expression compared to fructose (*P <* .01) and water controls (*P <* .05) (Figure 1(D)). The mRNA expression of *Vegf*, *Vegfr2*, *Ace1*, and *eNos* was similar in fructose and water controls. Exposure to BPA did not change the cardiac mRNA expression of the other genes included in the study (listed in Table 2) compared to fructose or water controls.

### BPA increased the mRNA expression of eNOS, ACE1, IL-8, and NFκβ in human cardiomyocytes

**Figure 2.**
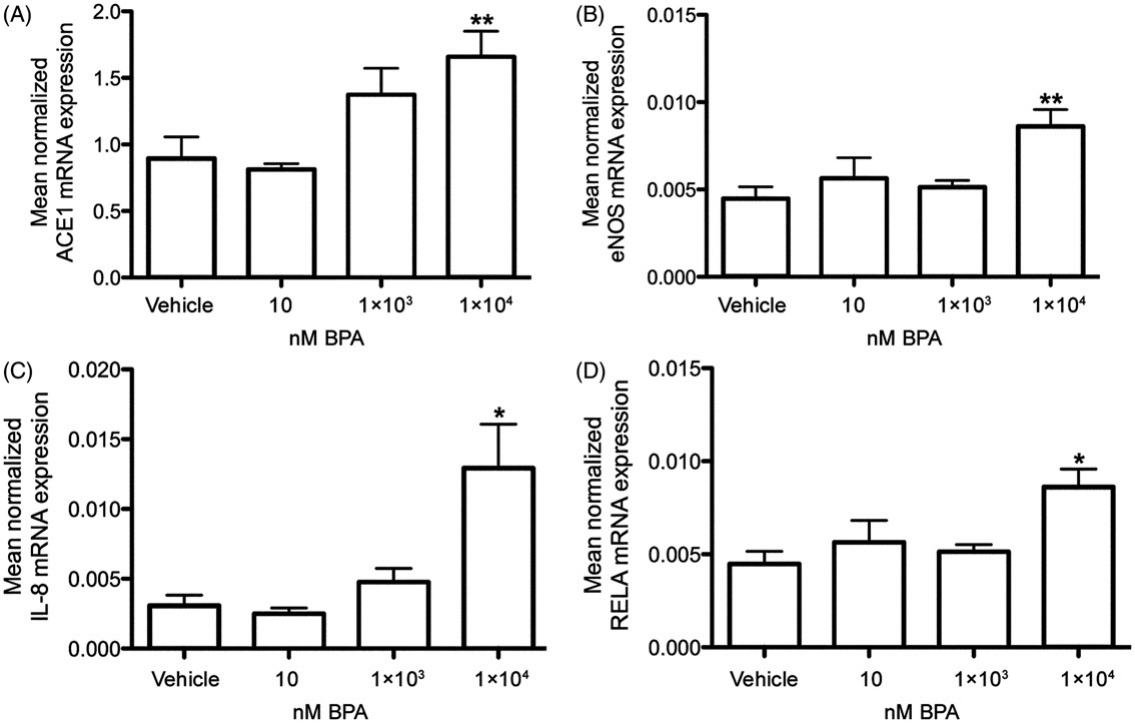
*In vitro* effects of BPA exposure on *ACE1*, *eNOS*, *IL-8*, and *NFκβ* mRNA expressions in primary human cardiomyocytes. Treatment of human cardiomyocytes with 1 × 10^4^ nM BPA for 6 hours increased the mRNA expression of *ACE1* (A), *eNOS* (B), *IL-8* (C), and *NFκβ* (RELA) (D), compared to vehicle controls. Each bar represents mean normalized mRNA expression ± SEM of three cell cultures, each analyzed in three replicates. *P < .05, **P < .01, compared to vehicle controls.

Six-hour incubation of HCMs with 1 × 10^4^ nM BPA increased the mRNA expression of *ACE1* (Figure 2(A)) and *eNOS* (Figure 2(B)) (*P <* .01, respectively) and also of the inflammatory markers *IL-8* (Figure 2(C)) and *NFκβ* (Figure 2(D); *RELA*) (*P <* .05, respectively) compared to vehicle-treated cells (Figure 2). Exposure to BPA did not change the mRNA expression of the other markers for cardiovascular function or the BPA target receptors compared to vehicle controls, examined in the present study (Table 2).

## Discussion

The results of the present study demonstrated that oral low-dose BPA exposure of rats from preadolescence to adulthood increased the mRNA expression of genes related to angiogenesis (*Vegf* and *Vegfr2*), a gene related to vasoconstriction (*Ace1*), and a gene related to endothelial dysfunction (*eNos*), in the heart. The present study also revealed that exposure to 10 μM BPA induced increased mRNA expression of *eNOS* and *ACE1* as well as mRNA expression of the inflammatory markers *IL-8* and *NFκβ* in cultured human cardiomyocytes. The effects of estrogens on endothelial cells are well described, whereas the effects on cardiomyocytes are less known. However, recent studies suggest that estrogens may regulate cardiac metabolism, attenuate myocardial apoptosis, and modulate myocardial hypertrophy in cardiomyocytes (36). Although cardiomyocytes dominate the volume of the myocardium, the number of endothelial cells exceeds the number of cardiomyocytes by approximately three to one (37). We have previously reported that cultured primary human endothelial cells exposed to BPA (1 nM to 1 μM) showed increased mRNA expression of *VEGF*, *VEGFR2*, *ACE1*, and *eNOS* (20). Hence, the present data suggest that BPA can increase expression of genes that are involved in the regulation of angiogenesis and vascular tone in rat heart as well as in human cardiomyocytes and endothelial cells. However, human endothelial cells seem to be more susceptible to BPA exposure than cultured human cardiomyocytes, and the effects of BPA in cardiomyocytes are restricted to *ACE1* and *eNOS*. Thus, BPA-induced changes in *VEGF*, *VEGFR2*, *ACE1*, and *eNOS* in the rat heart are more likely to be related to an effect of BPA on cardiac endothelial cells than cardiomyocytes. In a recently published report based on animals from the same *in vivo* rat study, it was shown that the myocardial proteome was altered after oral exposure to BPA and fructose. Proteins involved in, for example, fatty acid transport and oxidation, ROS generation, and structural integrity were altered, demonstrating that the combined exposure to BPA and fructose may induce distinct changes in the rat myocardium (29).

The present *in vivo* study demonstrated that long-term oral exposure of rats to ≥5 μg BPA/kg bodyweight/day increased cardiac mRNA expression of *Vegf* and *Vegfr2* mRNA expression, whereas higher doses of BPA did not change the *Vegfr2* mRNA expression. This is in line with our previous *in vitro* study, where we demonstrated that 1 nM BPA induced a stronger response on *VEGFR2* mRNA in cultured human endothelial cells compared to 1 × 10^3^ nM BPA (20). VEGF is known to be up-regulated under certain pathological conditions (24), and VEGF-driven angiogenesis can contribute to progression of atherosclerotic plaques (26). VEGF is also known to up-regulate *ACE1* mRNA expression in human endothelial cells, and angiotensin II up-regulates *Vegf* mRNA expression in rat cardiac endothelial cells (38,39). The present study revealed that BPA increased the *ACE1* mRNA expression in rat heart following oral exposure as well as *in vitro* in cultured human cardiomyocytes, suggesting that the BPA-induced mRNA expression of *ACE1* and *VEGF* in cardiac tissues may be associated events. Further, ACE1 is responsible for the formation of the vasoconstrictor angiotensin II, which affects blood pressure and is strongly involved in cardiovascular disease development (27,28).

VEGF is known as a potent inducer of *eNOS* mRNA expression in endothelium (40). The present study also revealed that oral exposure to BPA increased the mRNA expression of *eNos* (*Nos3*) in the rat heart, as well as in cultured human cardiomyocytes exposed to BPA. Nitric oxide derived from eNOS can contribute to angiogenesis and inflammation in the development and rupture of atherosclerotic plaques (41). Cultured human cardiomyocytes exposed to 1 × 10^4^ nM BPA also exhibited an increased mRNA expression of the cytokine *IL-8* and the transcription factor *NFκβ*, which are known as key factors driving inflammatory processes contributing to development of disease (42).

VEGF and ACE1 are also known as estrogen responsive genes in cardiovascular cells and tissues (21,22), and the estrogen-mimicking mode of action of BPA (11) may mediate the effects on ACE1 and VEGF. The observed effects of BPA on genes regulating angiogenesis and vascular tone in the rat heart may be due to a direct effect of BPA on target receptors in the heart. Indeed, mRNA expression of the BPA target receptors *Erα*, *Gpr30*, and *Errγ* was detected in all animals included in the present study, and *Gpr30* was detected in 6 of 20 animals. Notably, the qRT-PCR analysis did not indicate that oral exposure to BPA significantly changed the mRNA expression of the BPA target receptors in the rat heart.

The daily human intake of BPA is estimated to be around 1 μg/kg bodyweight/day (43). However, some epidemiological and experimental findings indicate that this is an underestimation of the true human exposure (5). The experimental design of the *in vivo* study was based on the estimated human exposure levels and the current US Food and Drug Administration (FDA) oral reference dose (RfD = 50 μg/kg bodyweight/day). Exposure of rats to BPA at 0.025 μg/mL, 0.25μg/mL, or 2.5 μg/mL in their drinking water resulted in an average intake of 5.0 μg/kg bodyweight/day, 53.5 μg/kg bodyweight/day, and 476 μg/kg bodyweight/day, respectively (Table 1). The lowest dose is about five times higher than the estimated average human intake, and the medium-high dose is similar to the current FDA RfD (43,44). Recently, the European Food Safety Authority (EFSA) has suggested a temporary RfD of 4 μg/kg bodyweight per day (45). Inter-species studies have suggested that BPA pharmacokinetics in women is similar to that in female monkeys and mice (46), indicating that the rodent animal model is relevant for the study of clearance of unconjugated BPA, and that the correlation between external and internal exposure is similar in rodents and humans. Further, the F344 rat used in the present study has been shown to excrete a larger portion of circulating BPA via the kidneys and urine, similar to human excretion (47). Moreover, the F344 rat has been reported to be more sensitive to endocrine disruption following BPA exposure compared with the commonly used Sprague-Dawley (S-D) rat (48,49). In the present study the rats were also given a modest dose of fructose (5%) in their drinking water to mimic a modern dietary pattern associated with the development of cardiovascular disease (30). However, the obtained data did not indicate that exposure to fructose alone induced any significant effects on markers for cardiovascular function in the rat heart.

In conclusion, the results of the present study demonstrate that low-dose exposure of rats to BPA, from preadolescence to adulthood, up-regulated the expression of genes that control angiogenesis and vascular tone in cardiac tissues. The BPA-induced increase of gene expressions in rat heart is in line with the BPA-induced changes in gene expression in cultured human cardiomyocytes and human endothelial cells (20), and is in concordance with a recently published report showing an altered cardiac proteome in BPA and fructose-exposed animals (29). Some of the observed alterations of gene expression in BPA-exposed rats have also been reported to be associated with coronary artery disease in humans, eventually leading to myocardial infarction. Thus, the observed changes in gene expression in BPA-exposed rats and in cultured human endothelial cells and cardiomyocytes may play a key role for the associations between BPA exposure and cardiovascular disease previously reported in epidemiological studies.
